# SiamMFC: Visual Object Tracking Based on Mainfold Full Convolution Siamese Network

**DOI:** 10.3390/s21196388

**Published:** 2021-09-24

**Authors:** Jia Chen, Fan Wang, Yingjie Zhang, Yibo Ai, Weidong Zhang

**Affiliations:** 1 National Center for Materials Service Safety, University of Science and Technology Beijing, Beijing 100083, China; b20160399@xs.ustb.edu.cn (J.C.); wangfan@xs.ustb.edu.cn (F.W.); zhangyingjie@xs.ustb.edu.cn (Y.Z.); ybai@ustb.edu.cn (Y.A.); 2 Southern Marine Science and Engineering Guangdong Laboratory, Zhuhai 519082, China

**Keywords:** visual object tracking, geometric characteristics, Siamese network, manifold features

## Abstract

Visual tracking task is divided into classification and regression tasks, and manifold features are introduced to improve the performance of the tracker. Although the previous anchor-based tracker has achieved superior tracking performance, the anchor-based tracker not only needs to set parameters manually but also ignores the influence of the geometric characteristics of the object on the tracker performance. In this paper, we propose a novel Siamese network framework with ResNet50 as the backbone, which is an anchor-free tracker based on manifold features. The network design is simple and easy to understand, which not only considers the influence of geometric features on the target tracking performance but also reduces the calculation of parameters and improves the target tracking performance. In the experiment, we compared our tracker with the most advanced public benchmarks and obtained a state-of-the-art performance.

## 1. Introduction

As a general object tracker, visual target tracking is one of the most important research contents in computer vision and has been widely concerned by more and more scholars. Visual object tracking technology has been widely used in security, video surveillance, and robot vision [1,2]. Although many high-performance trackers have been proposed in recent years, visual object tracking is still facing great challenges such as occlusion, deformation, and blur. Therefore, designing a high-performance tracker has very important research significance.

Recently, with the rise of deep learning convolutional neural networks, a growing number of scholars [3,4] have applied it to the field of object tracking and achieved good performance, of which Siamese network architecture is the most widely used. Siamese network framework can be regarded as a template target matching problem. The tracking task is realized by learning the similarity between a template branch and search branch, and the region with the highest similarity score is considered as the tracking object. Although the Siamese network tracker led by the SINT [5] algorithm achieved good results at the time, it was limited by the strict translation invariance restriction of the backbone network AlexNet [6], and the AlexNet with a shallower network depth was still used in the feature extraction stage. Although the performance of the later Siamese networks such as SiamRPN [7] has been greatly improved, the backbone network still adopts AlexNet.

Obviously, the Siamese network object tracker based on deep learning is mainly dedicated to the exploring and exploration of image semantic information and has achieved gratifying results. However, when the object is challenged by rapid deformation, the rich semantic information will also be subject to certain restrictions. Based on this, we increase the exploration of manifold information in the network structure. In this work, we propose a widely accepted manifold space hypothesis based on the concept that high-dimensional data is locally smooth when embedded in the manifold space. This hypothesis proves that when the semantic information is not enough to fully express the object characteristics, the manifold attribute can be used as a supplement to reflect the object characteristics more comprehensively. In terms of visual object tracking, we can regard a continuous video image sequence as different points in the manifold space, and objects with similar appearance characteristics should have the shortest distance in the embedded manifold space. In the manifold space, we use the distance between different points to express the degree of similarity of the geometric structure of the object, so that the manifold characteristics of the object can be observed.

In this paper, we combine the semantic information and geometric attributes of the object to propose a novel anchor-free design based on a Siamese network visual object tracker. We embed manifold components in the Siamese network structure to effectively combine the semantic information of objects with manifold features. By exploring the characteristics of the object, the Gaussian Mixture Model (GMM) is used to construct the manifold sample pool to make the manifold template more robust. In order to better apply manifold components to the network structure, we designed a deep architecture based on SiamRPN++, which adds a novel manifold template branch in parallel. In addition, in the semantic branch structure, we use an anchor-free tracker, which greatly reduces the number of parameters and computational complexity, making the tracker more robust. Finally, in order to verify the performance of our algorithm, we compared our proposed tracker with the state-of-the-art trackers on the OTB, GOT-10K, and UAV123 public benchmarks.

The main contributions of this work are as follows:

(1) Based on the manifold feature assumption of image data, an end-to-end so-called Siamese classification and regression framework for visual target tracking is proposed. The framework integrates the manifold sample branches of objects and inherits the advantages of semantic information and geometric attributes of objects.
(2) The proposed tracker is both anchor and proposal free, which greatly reduces the design of hyper-parameters and the influence of human factors, and makes the calculation more simple and fast, especially in training.
(3) Compared with the state-of-the-art trackers, our proposed algorithm SiamMFC has obtained a competitive advantage on three public benchmark datasets.

## 2. Manifold Fully Convolutional Siamese Networks

In this section, we will mainly introduce the Siamese network tracker based on the anchor-free design, and then we propose the manifold network to improve the tracking performance of the tracker.

### 2.1. Siamese Network Based on Anchor-Free

Siamese neural network refers to two or more neural network structures of the same subnet with the same parameters and weights, in which the input is two pictures, and the output is the similarity score between them. Visual target tracking regards tracking as a template matching problem, divides the video sequence into template branch and search branch, and finds the tracked object through the similarity measurement of the search branch on the template branch. The tracked object is the place with the highest similarity score. The tracking process can be expressed by Equation (1):

(1) $$f\left(z,x\right)\;=\;\varphi \left(z\right)\;*\;\varphi \left(x\right)\;+\;b\ell$$

where φ denotes the cross-correlation operation, and bℓ indicates that the score map for each position is not a single score value.

SiamRPN++ [8] has made a deep analysis of Siamese networks and found that the tracking accuracy is reduced because of the destruction of translation invariance in AlexNet [6] network when using deep networks. Therefore, the author proposes a simple and efficient sampling strategy to break the limitation of space invariance and successfully train the Siamese tracker with deep network ResNet-50 [9].

### 2.2. Manifold Learning Background

#### 2.2.1. Glassman Manifold

The advantages of smoothness and differentiability of Grassmann manifolds [10] allow us to use them to derive geometric properties of image sets. The Grassmann manifold is all linear subspaces of a given position number in a vector space V. $C^{N}$ is an N-dimensional complex Euclidean vector space. The Grassmann manifold $G\left(k,n\right)$, k<n, refers to the set of all k-dimensional subspaces in ${\mathbb{R}}^{n}$. A more mathematical representation is $G_{n,k}=O\left(n\right)∕O\left(k\right)\times O\left(n-k\right)$, where $O\left(n\right)$ is determined by orthogonal group quotient space composed of n×n orthogonal matrices [11].

Supposing that there is a point p in $G\left(k,n\right)$, and its n×k-dimensional orthogonal basis matrix is represented by U, and the full basis matrix in ${\mathbb{R}}^{n}$ is represented by Q (where $Q=[U|U_{⊥}]$ and $U^{T}U_{⊥}=0$). Then, the entire equivalence class can be expressed by matrix $U_{n\times k}$ as:

(2) $$\left[U\right]=\left\{UY_{k}:Y_{k}\in Q\left(k\right)\right\}$$

Meanwhile, the entire equivalence class representation of the Grassmann manifold can also be obtained in the quotient space:

(3) $$\left[Q\right]=\left\{Q\left(\begin{array}{cc} Y_{k} & 0\\ 0 & Y_{n-k} \end{array}\right):\left(\begin{array}{c} Y_{k}\in O_{\left(k\right)}\\ Y_{n-k}\in O_{\left(n-k\right)} \end{array}\right)\right\}$$

#### 2.2.2. Geodesics

**Figure 1:**
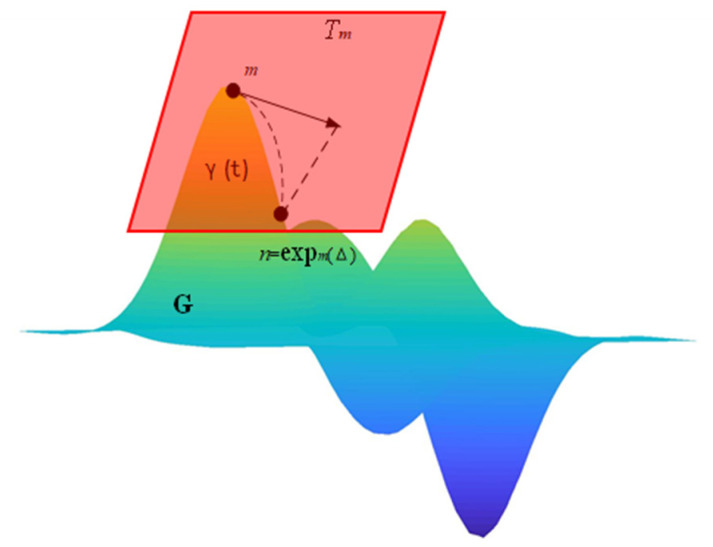
Visualization of 2-D manifold G embedded in $R^{3}$. $T_{m}$ is the tangent plane at point m, and $\gamma \left(t\right)$ is the geodesic distance between any two points m and n on the manifold space.

The geodesic is the curve with the shortest distance between two points in manifold space. In Euclidean space, the shortest path between two points is a straight line, and the shortest path between two points on a sphere is the arc length connecting the two points. For a Grassmann manifold, the distance between two points on the manifold is a geodesic, the calculation process is shown in Figure 1.

Suppose that m and n are two points in the popular space, and $T_{m}$ is the tangent plane between the point m and the manifold surface, which is composed of all the tangent vectors Δ at this point. Then the Grassmann manifold geodesic of the curve $\delta \left(t\right)=Y_{t}$ can be expressed by the following differential equation:

(4) $$\ddot{Y_{t}}+Y_{t}\left({\left(\dot{Y_{t}}\right)}^{T}\dot{Y_{t}}\right)=0\;$$

whose non-European canonical solution is:

(5) $$Y\left(t\right)=Y\left(0\right)expAt$$

where $A=\left(\begin{array}{cc} 0 & -B^{T}\\ B^{T} & 0 \end{array}\right)$ is n-by-n skew-symmetric and B is (n − p)-by-n skew-symmetric. Given Equation (4), under the premise of initial conditions $U\left(0\right)\;=\;U$ and $U\left(0\right)\;=\;H$, a simple method of calculating the geodesic is:

(6) $$Grassmann\;geodesics\;U\left(t\right)\;=\;\left(\begin{array}{cc} UV & R \end{array}\right)\left(\begin{array}{c} \cos\sum_{t}\\ \sin\sum_{t} \end{array}\right)V^{T}$$

the R in the formula represents the compact singular value decomposition $\left(SVD\right)$ of H.

### 2.3. Manifold Siamese Network Based on Anchor-Free

#### 2.3.1. Overall Network Structure

**Figure 2:**
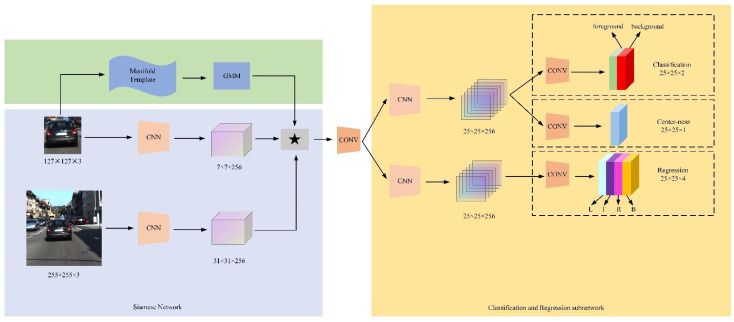
The pipeline of the proposed SiamMFC tracker, where the left side is the semantic feature and geometric feature extraction sub-network, and the right side is the classification and regression sub-network.

SiamRPN++ employs a Siamese network structure to explore the rich semantic information of objects and has achieved good performance in visual object tracking. However, in challenges such as the fast motion of objects, the potential geometric features of the objects can make our algorithm performance more robust, so we add manifold branches on the basis of the Siamese network to better explore the geometric features of objects, as shown in Figure 2. Among them, 255 × 255 × 3 is the search image, and the 3 here refers to the three color channels of r, g, and b of the image.

The network structure after joining the manifold branch can be expressed as:

(7) $$f\left(z,x\right)\;=\;\left[G\left(z\right)⊕\varphi \left(z\right)\right]\;*\;\varphi \left(x\right)\;+\;b\ell$$

Here, *b* represents the manifold feature extracted from the template branch and ⊕ represents the fusion operation of the semantic feature and geometric feature of the template branch.

In the manifold template branch, the template image passes through the manifold sample pool to extract geometric features and then merges with the semantic information extracted by the template branch and the search branch through the backbone network ResNet50 to obtain 25×25×256 feature maps, which pass through two conv layers performing classification and regression of objects, respectively. Different from Siamsrpn++, in order to more accurately reflect the score of the classification result, the center-ness branch is introduced in the classification branch, and the classification score is weighted by the score, away from the center of the object the closer the point is, the higher the score is. In the regression branch, the distance between the anchor point and the four edges is used to return the accurate position information.

#### 2.3.2. Manifold Template Branch

**Figure 3:**
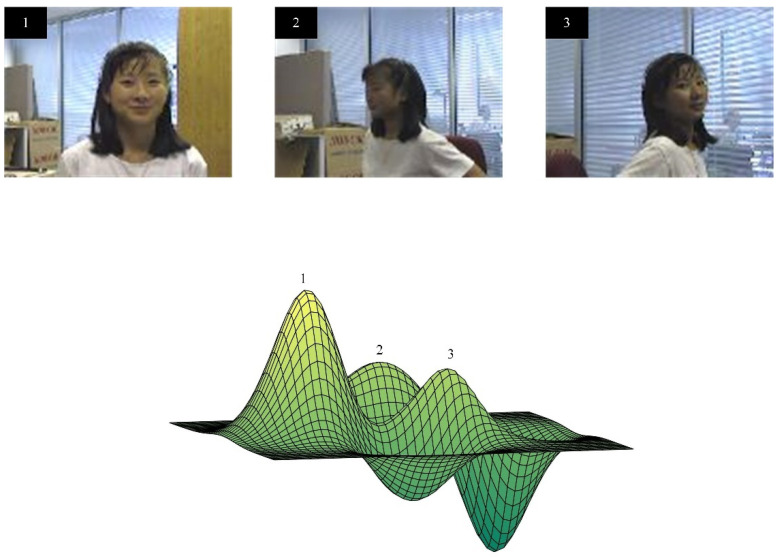
Different frames of the same target in a continuous image sequence correspond to different points on the Grassman manifold.

In the tracking process, if we start sampling a new sample dataset, it will bring complex and redundant calculations. However, almost all sampling strategies at this stage are similar samples containing the same semantic information, which seriously affects our tracking speed. In order to eliminate and avoid this phenomenon, and to better achieve a compact description of the data, we introduce a manifold sample pool in the Siamese network. In a given video sequence, the visual object in each frame can be regarded as a point on the Grassmann manifold, and the similarity between adjacent frames can be represented by a geodesic line between two points. The schematic diagram is shown in Figure 3.

In this work, because the base matrix U is controlled by the dominant feature vector of the image, it can well reflect the geometric characteristics of the sample, so we use the base matrix to describe the performance of the objects in the video sequence on the manifold. Suppose Z is an observation matrix constructed from a given video sequence Q:

(8) $$Z=[\tilde{z_{1}},\tilde{z_{2}},\tilde{z_{3}}\ldots {\tilde{z_{Q}}]}^{T}$$

Here, z represents the vector corresponding to each frame of image in the video sequence, $l=1/Q\sum_{i=1}^{Q}z_{i}$ is the average vector, and $\tilde{z_{i}}=z_{i}-l$ represents the average observation value extracted from the *i*-th frame image. The sample covariance matrix $C=ZZ^{T}$ can generate k principal eigenvectors from the basic matrix $U\in {\mathbb{R}}^{n,k}$. There are many methods to calculate the main eigenvalues. We consider the SVD decomposition method from the perspective of calculation efficiency as follows:

(9) $$\tilde{U}\tilde{D}\tilde{V}=SVD\left(Z\right)$$

Here, the sample U in the manifold space of each frame of the video sequence can be obtained by k principal vectors $\tilde{U}$. Then we can construct the manifold sample pool p and manifold sample feature t in the entire video sequence.

In order to more comprehensively reflect the contribution of different geometric features in the manifold sample pool to the continuous video sequence, we employ Gaussian Mixture Model(GMM) in probability model estimation to estimate the sample weight to reflect the different geometric attributes presented by different weight samples, whose standard expression formula of this model is as follows:

(10) $$p_{M}\left(x\right)=\overset{k}{\underset{i=1}{\sum }}\alpha_{i}N\left(x;\mu_{i};\sum_{i}\right)$$

where k represents the total number of samples, each sample corresponds to a Gaussian distribution, $\mu_{i}$ and $\sum_{i}$ are the parameters of the i-th sample, and $\alpha_{i}$ represents the sample weight. In this work, we convert the covariance matrix C to the identity matrix I in order to reduce the computational cost of high-dimensional samples.

In the online update of GMM components, in order to reflect the effectiveness and convenience of the algorithm, we apply the simplified algorithm proposed by Declercq and Piater [12] to replace the classic expectation maximization (EM) iterative optimization algorithm. Assuming that the new sample given in the initial stage is $\omega_{j}$, the parameters of the corresponding new Gaussian component n are $\alpha_{n}=\gamma ,\;\mu_{n}=\omega_{j}$, respectively. When simplifying and updating the GMM, compare the existing number of components with the set maximum value k of the number of components. If the sample weight $\alpha_{i}$ is less than the set threshold $K_{max}$, then the sample model does not meet the requirements. If the sample weight $\alpha_{i}$ is greater than the set threshold $K_{max}$, the two closest samples p and q in the manifold sample pool are merged into a new sample s. The specific calculation process is expressed by the following formula:

(11) $$\left\{\begin{array}{c} \alpha_{s}=\alpha_{p}+\alpha_{q}\\ \mu_{s}=\frac{\alpha_{p}\mu_{p}\;+\;\alpha_{q}\mu_{q}}{\alpha_{p}\;+\;\alpha_{q}} \end{array}\right.$$

In particular, the distance between p and q needs to be calculated using the geodesic distance in the manifold space. Since the calculation process of GMM is a two-step nested loop, which only includes a path search and a merge operation, the algorithm embodies the advantage of fast calculation speed.

#### 2.3.3. Siamese Sub-Network and Classification Regression Sub-Network Branch

Siamese Sub-network: The semantic branch employs the classic fully convolutional Siamese network, namely the template branch Z and the search branch X. As the shallow neural network has good visual attributes, which can extract good position information, and the deep network can extract rich semantic information, which has a good discriminating effect, and both are essential to the improvement of tracking accuracy. Therefore, the backbone network during feature extraction applies the modified ResNet-50 that aggregates multiple layers of features. In order to locate the object more accurately, we perform multi-level feature extraction in the last three residuals of ResNet-50 to achieve hierarchical aggregation, and the output features are $F_{3}\left(x\right)$, $F_{4}\left(x\right)$ and $F_{5}\left(x\right)$, respectively. Since the outputs of these three modules have the same spatial resolution, Equation (12) can be used for direct addition during weighted fusion.

(12) $$\varphi \left(x\right)\;=\;cat\left(F_{3}\left(x\right),\;F_{4}\left(x\right),\;F_{5}\left(x\right)\right)$$

where $\varphi \left(x\right)$ and $F_{3-5}\left(x\right)$ both contain 256 channels. After the deep cross-correlation between the search branch and template branch, a multi-channel corresponding graph was obtained. The convolution dimension reduction of the response graph and the 1×1 convolution kernel was carried out to 3×256 channels, which significantly reduced the number of parameters and accelerated the calculation in the following stages. Finally, the response graph $R^{*}$ obtained from the template branch and search branch is applied to the subsequent classification regression subnetwork.

Classification Regression Sub-network: SiamRPN++ adopts the center of the anchor-based on multi-scale design as the corresponding position of the object on the search area, and applies these anchors to return to the true bounding box of the object. Although the performance of SiamRPN++ is already state-of-the-art, the anchor-based tracker not only needs lots of human experience, and the huge parameter design also increases the complexity of the calculation. In order to overcome the above problems, our network applies anchor points to classify and regress objects.

There are two subtasks in the classification-regression sub-network, one is the classification task used to distinguish the foreground and the background, and the other is the regression sub-network used to obtain the precise position of the object. In particular, in order to more accurately reflect the accuracy of different anchor points for the return position of the object bounding box, we proposed a center-ness branch in the classification subtask. The higher the score, the closer to the object center point, the more accurate the return position. The response map $R_{w\times h\times m}^{*}$ obtained by the Siamese network after feature extraction passes through the classification branch and the regression branch to obtain the feature maps of $A_{w\times h\times 2}^{cls}$ and $A_{w\times h\times 4}^{reg}$, respectively, where *h* and *w* represent the height and width of the feature maps, respectively. For each image data, $A_{w\times h\times 2}^{cls}$ is a two-dimensional vector representing the score of the foreground and background, and $A_{w\times h\times 4}^{reg}$ is a four-dimensional vector $t\left(i,j\right)\;=\;\left(l,t,r,b\right)$ representing the position of the regression bounding box.

Since the proportion of foreground and background in the search area is not very large, the loss function does not involve the imbalance problem. Hence, the cross-entropy loss and IOU loss functions are employed for classification and regression, respectively.

For any point $\left(i,j\right)$, $\left(x,y\right)$ represents its corresponding position coordinates, assuming that $\left(x_{1},y_{1}\right)$ and $\left(x^{*},y^{*}\right)$ represent the coordinates of the left-top corner and the right-bottom corner of the ground truth bounding box, respectively. Then the regression target at $A_{w\times h\times 4}^{reg}\left(i,j,:\right)$ can be calculated by the following formula:

(13) $${\tilde{t}}^{0}{}_{\left(i,j\right)}=\tilde{l}=x-x_{1},{\tilde{t}}^{1}{}_{\left(i,j\right)}=\tilde{t}=y-y_{1}\;{\tilde{t}}^{2}{}_{\left(i,j\right)}=\tilde{r}=x^{*}-x,{\tilde{t}}^{3}{}_{\left(i,j\right)}=\tilde{b}=y^{*}-y$$

With the formula t of IOU between the ground-truth bounding box and the predicted bounding box, the loss function of the regression task is calculated as follows:

(14) $$L_{reg}=\frac{1}{\sum 𝕝\left({\tilde{t}}_{\left(i,j\right)}\right)}\sum_{i,j}𝕝\left({\tilde{t}}_{\left(i,j\right)}\right)L_{IOU}\left(A^{reg}\left(i,j,:\right),{\tilde{t}}_{\left(x,y\right)}\right)$$

Inspired by [13], the calculation of $L_{IOU}$ adopts the same loss method as Unitbox, and $𝕝\left(\cdot \right)$ is calculated by the following formula:

(15) $$𝕝\left({\tilde{t}}_{\left(i,j\right)}\right)=\left\{\begin{array}{c} 1\;if\;{\tilde{t}}^{k}{}_{\left(i,j\right)}>0,k=0,1,2,3\\ 0\;otherwise \end{array}\right.$$

Although anchor point prediction reduces the number of parameters and calculation, after many bounding box predictions, it is found that the prediction effect of the 78uposition far away from the center of the object is not good, which directly leads to the degradation of the tracker’s performance. To this end, inspired by FCOS [14], we added a center-ness feature map $A_{w\times h\times 1}^{cen}$ in the classification branch to eliminate low-quality prediction bounding boxes. The score map $C\left(i,j\right)$ in $A_{w\times h\times 1}^{cen}$ is between 0 and 1, which can be calculated by the following formula:

(16) $$C\left(i,j\right)=𝕝\left({\tilde{t}}_{\left(i,j\right)}\right)\;*\;\sqrt{\frac{\min\left(\tilde{l},\tilde{r}\right)}{\max\left(\tilde{l},\tilde{r}\right)}\times \frac{\min\left(\tilde{t},\tilde{b}\right)}{\max\left(\tilde{t},\tilde{b}\right)}}$$

The value of $C\left(i,j\right)$ reflects the distance between the position point (*x*, *y*) and the center of the object. The higher the value of $C\left(i,j\right)$, the closer the anchor point (*x*, *y*) is to the center of the object, and the better the prediction effect. If the anchor point is in the background, then $C\left(i,j\right)$ is set to 0. The calculation formula of the loss function of the center-ness score is:

(17) $$L_{cen}=\frac{-1}{\sum 𝕝\left({\tilde{t}}_{\left(i,j\right)}\right)}\sum_{𝕝\left({\tilde{t}}_{\left(i,j\right)}\right)==1}C\left(i,j\right)\;*\;logA_{w\times h\times 1}^{cen}\left(i,j\right)+(1-C\left(i,j\right)\;*\;log\left(1-logA_{w\times h\times 1}^{cen}\left(i,j\right)\right)$$

The overall loss function of the network is:

(18) $$L=L_{cls}+\lambda_{1}L_{cen}+\lambda_{2}L_{reg}$$

It is particularly noted that $\lambda_{1}$ and $\lambda_{2}$ are weight hyperparameters that balance center-ness loss and the regression loss, which are set to $\lambda_{1}$ = 1 and $\lambda_{2}$ = 3 empirically.

## 3. Experiments

### 3.1. Implementation Details

The proposed SiamMFC was trained on two 1080ti GPUs using pytorch for 7 days. The training phase uses four datasets of COCO [15], ImageNet DET [6], ImageNet VID [6], and YouTube-BB [16], and the template branch and search branch images use 127 and 255 pixel image pairs, respectively. The backbone network uses modified ResNet-50 to perform convolution operations on the randomly initialized 1×1 convolutional layer with conv3, conv4, conv5 to reduce the feature dimension to 256. SiamMFC uses stochastic gradient descent (SGD) to optimize the training of the network. Considering the memory of GPU, the batch size is set to 48, and a total of 20 epochs are trained. The first 5 epochs are used to train the RPN branch with a learning rate of 0.001, and the learning rate of the next 15 epochs decays from 0.005 to 0.0005 in the form of exponential decay, which employs end-to-end training. A weight decay of 0.0005 and momentum of 0.9 are adopted throughout the training process.

### 3.2. Results on OTB

**Figure 4:**
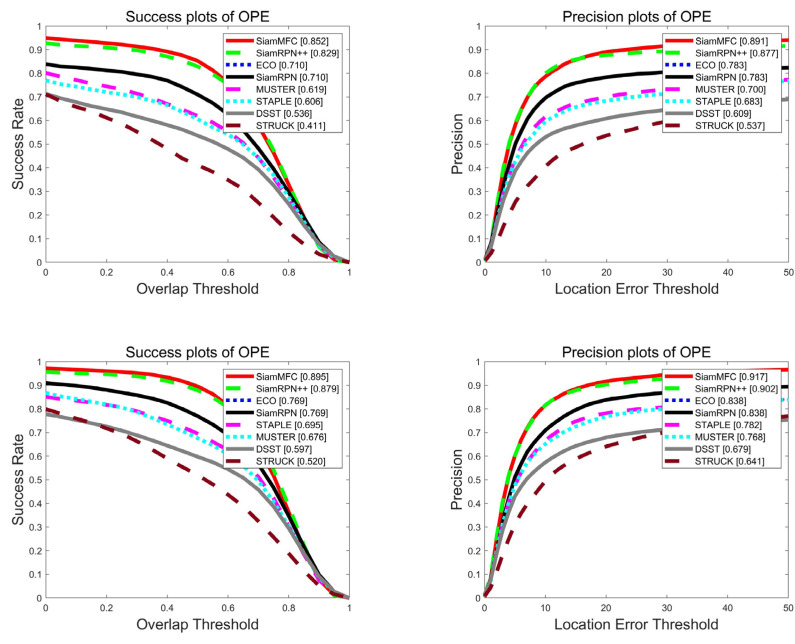
The overall performance comparison plot of the OTB, where the first row is OTB2013 and the second row is OTB2015.

***(1) Global Performance:*** The OTB dataset is a fair and robust evaluation benchmark platform proposed by Wu Yi [17], which is divided into two data sets, OTB50 and OTB100. Among them, OTB50 contains 50 video sequences, and OTB100 is composed of 100 video sequences including OTB50. This dataset has artificially labeled groundtruth and contains 25% grayscale datasets. Figure 4 shows the intuitive comparison between our proposed tracker algorithm SiamMFC and the state-of-the-art algorithms SiamRPN++ [8], ECO [18], MUSTER [19], STAPLE [20], STRUCK [21], DSST2 [22], and SiamRPN [7]. It can be seen that our algorithm ranks first, and the AUC performance exceeds the benchmark SiamRPN++ by 2.3% and 1.6% respectively.

**Figure 5:**
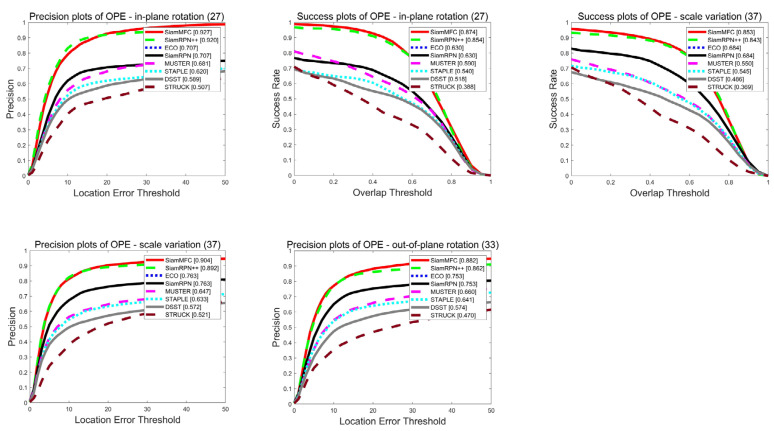
Performance comparison plot of eight trackers in different challenges of OTB2013.

In order to further demonstrate the advantages of our algorithm, we compared SiamMFC with SiamRPN++, ECO, MUSTER, STAPLE, STRUCK, DSST, and SiamRPN in the more challenging OTB50 video sequence. Figure 5 shows that the SiamMFC tracker ranks first with a competitive advantage in the challenges of in-plane rotation, out-of-view, and scale variation.

**Figure 6:**
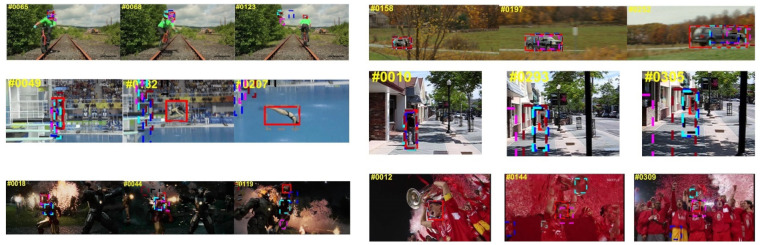
Visualization of the qualitative analysis results of SiamMFC and the remaining seven state-of-the-art trackers on the OTB dataset.

***(2) Qualitative Analysis:*** Figure 6 shows a comprehensive and intuitive qualitative comparison between the SiamMFC tracker and the seven state-of-the-art trackers mentioned above on several challenging video sequences. Sequence 1 is a young man riding a bicycle on a railroad track. Although the tracking effect of all the trackers was very good in the initial stage, after the young man turned around, only the tracking of SiamMFC was the most accurate. Sequence 2 is a moving car. After frequent occlusion and deformation of the car, although some trackers have good tracking results, because we adopt an anchor-free tracking strategy, only the SiamMFC tracker is the most compact. Deformation is one of the huge challenges in visual object tracking. After the divers in video 3 are flipped and deformed in the air, the SiamMFC shows a good tracking effect. In the three video sequences of 4, 5, and 6, the SiamMFC can still track the object well after the fast-moving, background clutter, and occlusion of the tracked object.

### 3.3. Results on UAV123

UAV-123 is a dataset composed of videos captured by low-altitude drones, which is essentially different from the video captured by OTB50 and other mainstream tracking datasets. A subset of the dataset is used for long-term air tracking, which contains a total of 123 video sequences and more than 110 k frames. All sequences in the dataset can be annotated with a manual box and can be easily integrated with the visual tracker benchmark, which contains all ground-truth boxes and attribute annotations of the UAV dataset.

In order to better verify the performance of our tracker, we compared SiamMFC on the UAV123 dataset with the benchmark algorithm SiamRPN++ and the state-of-the-art performance trackers SiamRPN, ECO, DaSiamRPN [23], and ECO-HC [18]. The comparison results are shown in Table 1, in which it can be clearly seen that our proposed SiamMFC ranks first in AUC score by 7% higher than the baseline SiamRPN++.

### 3.4. Results on GOT-10K

GOT-10k [24] is a large-scale target tracking dataset based on WordNet, which widely covers 560 types of common outdoor moving objects. The bounding boxes of the objects are all manually labeled, and the number of bounding boxes exceeds 1.5 million, which realizes the unified training of the depth tracker and stable evaluation. GOT-10k has the advantages of large-scale, general-purpose, single-sample learning, uniform training data, additional labeling, and effective evaluation.

We evaluated our proposed algorithm SiamMFC on GOT-10k and compared it with the six advanced trackers SiamFC, CCOT [25], ECO, MDNet [26], SiamRPN++, and SiamMFC, as shown in Table 2. The performance of our algorithm is highlighted in red.

### 3.5. Ablation Studies

As a kind of geometric information, manifold brings superior performance improvement to our tracking algorithm. In order to more clearly show the change of manifold characteristics, we conducted ablation research on the variation of our algorithm on the UAV-123 algorithm, as shown in Table 3. The results of using only ResNet-50 and manifold are not as high as the effective fusion index of the two.

## 4. Conclusions

In this work, we combine the rich geometric properties of objects with semantic information and propose a novel end-to-end visual object tracker, SiamMFC. In the manifold branch, the application of manifold learning and the Gaussian Mixture Model (GMM) to update the sample template is a good way to exploit the geometric attributes of the object. In the semantic branch, in addition to the modified ResNet-50 network in the feature extraction stage, the application of the anchor-free tracker idea in the classification and regression stage not only greatly reduces the amount of parameters and the rigidity of human experience, but also reduces the complexity of the calculation method, establishing a good foundation for the application of more practical scenarios. Compared with baseline algorithm SiamRPN++, our algorithm has a 2.3% higher success rate and 1.4% higher accuracy on challenging OTB50 datasets. The success rate on GOT-10K datasets is 3.7% higher than SiamRPN++. In future work, we will continue to explore the deeper potential of the geometric attributes and semantic information of objects, and apply them to visual target tracking.

## Figures and Tables

**Table 1:** Comparison results on UAV-123 benchmark.

	ECO-HC	SiamRPN++	SiamRPN	ECO	DaSiamRPN	SiamMFC
AUC (%)	0.506	0.613	0.527	0.525	0.586	0.620
P (%)	0.725	0.807	0.748	0.741	0.796	0.811

**Table 2:** Comparison results on GOT-10k benchmark.

Tracker	AO	$SR_{0.5}$	$SR_{0.75}$	Hardware	Language	FPS
SiamFC	0.374	0.404	0.144	Titan X	Matlab	25.81
CCOT	0.325	0.328	0.107	CPU	Matlab	0.68
CFNet [27]	0.293	0.265	0.087	Titan X	Matlab	35.62
SPM [28]	0.513	0.593	0.359	Titan XP	Python	72.3
BACF [29]	0.260	0.262	0.101	CPU	Matlab	14.44
MEEM [30]	0.253	0.235	0.068	CPU	Matlab	20.59
ECO	0.316	0.309	0.111	CPU	Matlab	2.62
MDNet	0.299	0.303	0.099	Titan X	Python	1.52
SiamRPN++	0.517	0.616	0.325	RTX 1080ti	Python	49.83
SiamMFC	0.554	0.668	0.413	RTX 1080ti	Python	51.13

**Table 3:** The results of the ablation experiment.

Component	UAV-123	
	AUC (%)	P (%)
SiamRPN++	0.613	0.807
SiamRPN++ (anchor-free)	0.617	0.809
SiamRPN++ and manifold	0.615	0.808
Ours	0.620	0.811

## Data Availability

Not applicable.
